# The Impacts of Shatamuli, Ashwagandha and their Combination on Growth Performance, Faecal Microbial Population and Blood Parameters in Sonali Chickens

**DOI:** 10.1002/vms3.70840

**Published:** 2026-02-07

**Authors:** Ayesha Siddika, Md. Ahsan Habib, Ummay Salma, Md. Nurul Amin, Sabbir Hossen Sabuz, Md Abdur Rahman Toha

**Affiliations:** ^1^ Department of Animal Science and Nutrition Faculty of Veterinary and Animal Science Hajee Mohammad Danesh Science and Technology University Dinajpur Bangladesh

**Keywords:** ashwagandha, blood parameters, growth performance, shatamuli, sonali chicken

## Abstract

**Background:**

Phytogenic feed additives have shown potential in enhancing nutrient utilization, improving performance and mitigating pathogenic challenges in Sonali chicken.

**Objective:**

The goal of the current study was to assess how Shatamuli (*Asparagus racemosus*) and Ashwagandha (*Withania somnifera*) affected the Sonali chickens' growth performance, blood parameters, carcass characteristics and faecal microbial population.

**Methods:**

96‐day‐old Sonali chicks were reared for 63 days and randomly assigned to four dietary groups with three replications and each contains eight birds where T_0_: Basal Diet (BD)/control, T_1_: BD + 0.5% Ashwagandha root powder, T_2_: BD + 1% Shatamuli root powder and T_3_: BD + 0.5% Ashwagandha root powder + 0.5% Shatamuli root powder were supplied. Body weight gains, feed efficiency and mortality were recorded. At 63 days, blood samples were collected for haematological test, birds were slaughtered to assess carcass traits and faecal samples were collected for microbial analysis.

**Results:**

The T_3_ group showed the highest (*p* < 0.01) weight gain and final body weight, followed by T_2_, T_1_ and T_0_ groups. The T_3_ group ingested a higher quantity of feed than the other groups. The T_3_ group had the best feed efficiency and the highest carcass, dressing percentage, breast meat, thigh muscle, drumstick, wings and gizzard weight. Blood analysis revealed lower cholesterol, triglycerides, LDL and glucose, with higher HDL and haemoglobin in the T_3_ group. In every treatment group, faecal bacterial counts showed a highly significant decline (*p* < 0.01), with the lowest in the T_3_ group. Economic analysis showed the highest net profit from the T_3_ group.

**Conclusion:**

Combined Shatamuli and Ashwagandha root powder significantly improved Sonali chickens' growth, health, and profitability.

## Graphical abstract

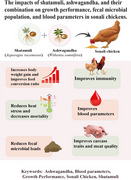

## 1 Introduction

The poultry industry is the largest segment of the global livestock sector and significantly contributes to the economy in transitioning nations by reducing poverty. Asia has recently taken the lead in global poultry markets, surpassing other regions (Kandeel et al. 2022). In Bangladesh, this industry accounts for nearly 22%–27% of the total supply of animal protein (Islam et al. 2019). The Sonali chicken, developed by crossing RIR with Fayoumi and closely resembling native chickens in appearance, is now widely reared because of its rapid growth, resilience to disease, lower mortality and higher profitability (Rahman et al. 1997). Synthetic drugs and growth stimulants for Sonali chickens have adverse health effects, necessitating the search for non‐therapeutic alternatives like inorganic acids, probiotics, prebiotics, botanicals and enzymes (Bhujbal et al. 2009). Phytogenic feed additives (PFAs) have recently gained popularity for enhancing performance by maintaining a healthy intestinal environment (Oso et al. 2019).

The tropical lily plant Shatamuli, known scientifically as *Asparagus racemosus*, is designated as the ‘Queen of Herbs’ and has been utilized since pre‐Vedic times. Its roots are mainly saponins, such as shatavarin I‐IV, the glycosides of sarsasapogenin. Shatamuli's nourishing, anti‐stress, adaptogenic, immunomodulatory, galactogenic and anabolic compositions and its performance‐enhancing ingredients make it a valuable component in many medicinal compositions. It nourishes and regenerates tissue while increasing vitality and strength. Antioxidant, anti‐diabetic, antifungal, antibacterial, antiviral, hepatoprotective, antiarthritic, anti‐inflammatory, antiperiodic, anti‐ulcerogenic, anti‐abortive, analgesic, astringent, emollient, cooling, nerve tonic are just a few of the many health benefits (Akhtar et al. 2024). *A. racemosus* (commonly known as Shatamuli) is well‐regarded for its nutritional and therapeutic benefits, and numerous studies have examined the effects of adding its root powder to broiler diets to improve growth performance (Kumar et al. 2019; Patil et al. 2024; Verma et al. 2024).

*Withania somnifera*, generally referred to as Ashwagandha, is another herbal supplement with diverse biological functions, such as promoting growth, regulating lipid metabolism, alleviating stress, improving antioxidant defence, adjusting immunological responses, lowering inflammation and supporting haematological parameters, including haemoglobin and platelet levels (Salem et al. 2022). Evidence also suggests that Ashwagandha may influence cortisol secretion, improve physical performance and mitigate fatigue, while providing immunological benefits under stress conditions.

Although Shatamuli and Ashwagandha have been shown to possess pharmacological properties, their use in poultry, particularly in the Sonali breed, has not been thoroughly studied. Very little is known about the combined effects of these herbs in poultry, as most previous research has concentrated on them separately in broiler or layer breeds. Furthermore, no previous study has examined their combined (synergistic) effects or simultaneously assessed the gut microbiota, haematological parameters and growth performance in Sonali chickens. A potential natural substitute for traditional growth promoters, this study is unique in that it evaluates, for the first time, the effects of these two herbal adaptogens separately and in combination on various physiological and productive outcomes in Sonali chickens raised in Bangladesh.

## 2 Materials and Methods

The Animal Experiments Ethics Committee of Department of Animal Science and Nutrition, Hajee Mohammad Danesh Science & Technology University (HSTU/VAS/ASN/EA/020) and the Bangladesh Veterinary Council gave their approval for all the animal tests that were done. This action was taken with the intention of safeguarding the animals' welfare.

### 2.1 Experimental Site

Central Poultry Farm at Hajee Mohammad Danesh Science and Technology University (HSTU), Dinajpur, Bangladesh, was the experimental place where the research was conducted from 2 June 2024 to 4 August 2024. The blood plasma profile was analysed at Prof. Emdadul Haque Prani Sheba Kendra and Veterinary Diagnostic Center, Baliadangi, Thakurgaon, Bangladesh, and at the pathology laboratory under the Department of Pathology and Parasitology in HSTU. A faeces test was performed at the Microbiology lab, HSTU.

### 2.2 Experimental Design

The experiment was organized in a completely randomized design (CRD). In this trial, a total of 96 day‐old Sonali chicks, produced by hybridizing Rhode Island Red males and Fayoumi females, were obtained from Nahar Poultry and Hatchery Ltd. The chicks were brooded for seven days under standard management before being randomly distributed into four dietary treatment groups (T_0_, T_1_, T_2_ and T_3_), each consisting of three replications with eight birds. The experiment layout is illustrated in Table 1. The experimental treatments were as follows: T_0_ served as the control, T_1_ received feed supplemented with 0.5% Ashwagandha root powder, T_2_ was provided diet containing 1% Shatamuli root powder and T_3_ was fed a combination of 0.5% Ashwagandha plus 0.5% Shatamuli root powder.

**TABLE 1:** Layout of the experiment.

Replication	Dietary treatment				Total
	T_0_	T_1_	T_2_	T_3_	
R_1_	8	8	8	8	32
R_2_	8	8	8	8	32
R_3_	8	8	8	8	32
Total	24	24	24	24	96

*Note*: T_0_—Control diet without the inclusion of Shatamuli or Ashwagandha root powder.

T_1_—Diet supplemented with 0.5% Ashwagandha root powder.

T_2_—Diet supplemented with 1% Shatamuli root powder.

T_3_—Diet supplemented with 0.5% Shatamuli and 0.5% Ashwagandha root powder.

**TABLE 2:** Nutrient and ingredient composition of the basal feed.

Ingredients (%)	Starter (0–30 days)	Grower (31–63 days)
Metabolizable energy_min_ (kcal/kg)	2950	3000
Moisture_max_ (%)	12	12
Crude protein_min_ (%)	20	19
Crude fat_min_ (%)	4	4.5
Crude fibre_max_ (%)	4	4
Lysine_min_ (%)	1.2	1.1
Methionine_min_ (%)	0.45	0.40
Calcium_min_ (%)	1	0.90
Phosphorus_min_ (%)	0.48	0.45
Vitamins and minerals	As per the requirement	As per the requirement

*Source*: Nahar Poultry and Hatchery Limited, Bangladesh.

### 2.3 Collection of Experimental Feed and Additives

Basal feed was obtained from Nahar Poultry and Hatchery Limited (Table 2), Ramdubi Bazar, Dinajpur, Bangladesh. Feed was given in two phases: Starter and grower ration. The experimental feed additive for this study was Shatamuli and Ashwagandha root powder, which were purchased from the local market in Dinajpur (Mala Paint House, Maldahopotti).

### 2.4 Management Practices

In the experiment, the environmental conditions were meticulously regulated to promote optimal growth and health. The temperature was sustained at 35°C–33°C throughout the initial days and progressively decreased to 23°C–25°C following the brooding phase. Relative humidity was maintained at 60%–70%, and adequate ventilation was ensured to facilitate the circulation of fresh air and eliminate excess moisture and ammonia. During the first week, lighting was controlled at 23–24 h of light per day, which was subsequently reduced to approximately 16 h. Strict biosecurity procedures were followed to prevent disease, and clean, dry litter, 5–7 cm deep, was maintained throughout the period. Overall, these environmental conditions ensured favourable growth performance and welfare of the Sonali chickens. A chick's first seven days of life are the most crucial because this is when their immune system, digestive organs and capacity to regulate body temperature all develop quickly. Effective control of temperature, humidity and light is crucial for maintaining survival and promoting consistent growth. It also reduces stress and preserves standardization for reliable experimental outcomes. Intensive brooding is no longer necessary after seven days since Sonali chicks are stronger, feathered and less susceptible to temperature changes. Day‐old chicks were given glucose and vitamin C to prevent stress during transportation. To fight stress caused by high ambient temperature, water‐soluble vitamins, normal saline, electrolytes and vitamin C were supplied during the brooding phase. The chicks were vaccinated against Baby Chick Ranikhet Disease (BCRDV) (5 and 28 days) and Infectious Bursal Disease (IBD) (14 and 22 days) according to the Livestock Research Institute (LRI), Mohakhali, Dhaka.

### 2.5 Blood Collection and Separation of Blood

Approximately 3 mL of blood was obtained from a single bird per replicate through the wing vein utilising a sterile syringe and needle. Samples were placed in heparinized and non‐heparinized tubes and kept on ice. Centrifugation was used to separate the serum for 10 min at 3000 rpm, and it was sent to the Pathology Laboratory within 2 h for lipid profile analysis (cholesterol, HDL, LDL and triglycerides) and other blood parameters.

### 2.6 Determination of Total Cholesterol

Initially, using a micropipette, 10 µL of ready serum was transferred into each cuvette (1 cm light path). Each cuvette was then filled with 1000 µL of reagent, which was thoroughly mixed by shaking. The cuvettes were incubated for 5 min at 37°C prior to being placed in the spectrophotometer. Then, at a wavelength of 505 nm, each cuvette was placed in the spectrophotometer (Spectronic, Genesis 5, USA) against the blank reagent. Finally, the result was recorded from the display. The result was expressed in milligrams per decilitre (mg/dL).

### 2.7 Determination of Triglycerides (TG)

A spectrophotometer is used to measure the triglycerides in blood serum (Spectronic, Genesis 5, USA). The reagent was prepared for use, and all of the reagents were mixed correctly for the experiment in accordance with the company's (Reactivos GPL) instructions. The rest procedure is similar to the total serum cholesterol. The result was expressed in milligrams per decilitre (mg/dL).

$$\mathrm{Triglycerides}\;\left(\mathrm{mg}/\mathrm{dl}\right)=\mathrm{Abs}\;\mathrm{of}\;\mathrm{sample}/\mathrm{Abs}\;\mathrm{of}\;\mathrm{STD}\times \mathrm{Conc}.\;\mathrm{STD}$$

where Abs indicates absorbance and STD indicates standard deviation

### 2.8 Determination of High‐Density Lipoproteins (HDL)

The study involved mixing 50 µL of serum samples with 1,000 µL of reagents in a test tube, incubating at 37°C for 5 minutes, and then comparing the mixtures in a spectrophotometer (Spectronic, Genesis 5, USA) at a wavelength of 546 nm. Results were recorded and expressed in milligrams per deciliter (mg/dL).

### 2.9 Determination of Low‐Density Lipoprotein (LDL)

LDL concentration was calculated using the Friedewald equation:

$$\begin{array}{c} \mathrm{LDL}\;\left(\mathrm{mg}/\mathrm{dL}\right)=\mathrm{Total}\;\mathrm{serum}\;\mathrm{cholesterol}\\ -\left[\mathrm{Triglycerides}/5+\mathrm{HDL}\;\mathrm{cholesterol}\right] \end{array}$$

This formula estimates LDL by subtracting the sum of HDL cholesterol and one‐fifth of triglyceride concentration from the total serum cholesterol value.

### 2.10 Red Blood Cell (RBC) Count

The RBC count was determined using the hemocytometer technique. Anticoagulated blood was diluted at a ratio of 1:200 with Natt–Herrick's solution, and the mixture was introduced into a clean Neubauer counting chamber. After allowing the cells to settle, erythrocytes were counted under a light microscope at 400× magnification within five small squares of the central grid, adhering to standard boundary rules. The RBC concentration (cells/µL) was calculated using the formula:

RBC/μL=Numberofcellscounted×10,000

The final value was expressed as the mean of two replicate counts per microliter of blood.

### 2.11 Determination of Glucose

Three test tubes were identified as Blank (B), Standard (S) and Unknown (U). 1 mL of the working reagent was placed in each tube. After that, Blank was filled with 10 µl of distilled water. 10 µL of the standard solution was added to the standard tube, and 10 µL of serum was added to the test tube marked ‘Unknown.’ After that, the tubes were thoroughly mixed and incubated at 20–25°C for 10 min. Finally, a calorimeter set to 546 nm was used to measure light absorbance.

$$\begin{array}{ccc} & & \mathrm{Glucose}\;\mathrm{concentration}\;\left(\mathrm{mg}/\mathrm{dl}\right)\\ & & =\left(\mathrm{Unknown}\;\mathrm{absorbance}/\mathrm{Standard}\;\mathrm{absorbance}\right)\times 100 \end{array}$$

### 2.12 Evaluation of Carcass Characteristics

Three birds were randomly selected for slaughter in a replication, with feed withheld overnight. Sonalis were identified using leg band numbers and slaughtered using the Halal method. Following the evacuation of their blood, they were immersed in hot water (51°C–55°C) for 120 s to remove their feathers, the birds were cut into carcass parts to separate organs, including liver, spleen, heart, gizzard and meat yield.

### 2.13 Determination of pH and Drip Loss

The meat sample was ground, pH was recorded using a calibrated pH meter within one hour of slaughter, and breast muscle drip losses were measured after slaughter. Regular‐shaped muscle was cut into 20 g and stored in a 4°C refrigerator. After 24 h, samples were reweighed, and the difference in weight was used to calculate the drip loss.

### 2.14 Faecal Sample Collection and Microbial Load Enumeration

Faecal samples were aseptically collected from experimental birds before the trial began and on a weekly basis for three consecutive weeks. Each sample was suspended in 99 mL of phosphate‐buffered saline (PBS) or sterile normal saline. Serial dilutions (10^−^^1^ to 10^−^^1^ ^0^) were prepared by transferring 1 mL of the suspension into 9 mL of diluent in successive test tubes, discarding 1 mL from the final dilution. From the 10^−^^5^, 10^−^^7^ and 10^−^^9^ dilutions, 0.1 mL aliquots were spread in triplicate onto Plate Count Agar (PCA) plates. PCA was prepared by dissolving 23.5 g in 1 L of distilled water, which was then autoclaved at 121°C for 15 min. The solution was cooled to 55°C and then poured into Petri dishes. Plates were incubated at 37°C for 24 h, and colony‐forming units (CFU) were counted using standard methods. All microbial analyses were conducted at the HSTU Microbiology Laboratory.

### 2.15 Calculation

*Feed Intake (FI)*: Daily FI was determined by offering a predetermined amount of feed to each bird or group and weighing the residual feed at the end of the day. The actual amount of feed ingested by each bird was indicated by the difference between the feed that was provided and the feed that was left over.
*Feed Conversion Ratio (FCR)*: The FCR served as an indicator of how efficiently the birds converted feed into body mass. The computation involved dividing the total amount of feed ingested by the total amount of body weight gained over the same time period. A lower FCR value denotes higher feed efficiency.
*Live Body Weight (BW)*: Birds were individually weighed on a weekly basis using a digital scale to monitor growth performance. The weights of individual birds were recorded, and group averages were computed to evaluate overall growth patterns.
*Weekly Weight Gain (WWG)*: WWG was obtained by subtracting the bird's weight at the beginning of the week from its weight at the end of the week. This parameter indicated the rate of growth during each week of the trial.
*Mortality Rate (MR)*: Counting the number of birds that deceased during the trial period allowed for the assessment of the MR. It served as an indicator of the general health of the flock and was given as a percentage of the original bird population.

#### 2.15.1 Dressing Yield (%)

The weight of the carcass is divided by the weight of the living animal, and the result is multiplied by 100 to determine the percentage.

#### 2.15.2 Drip Loss (%)

It is calculated by deducting the meat sample's end weight from its initial weight, dividing that result by the initial weight, and then multiplying the result by 100.

#### 2.15.3 Colony Forming Unit (CFU/g)

It is counted by taking the number of colonies that grow on a plate, multiplying by the dilution factor and dividing by the volume of the sample that was plated.

### 2.16 Statistical Analysis

SPSS software (version 24.0) was used to analyse the data using a CRD. To evaluate the variations between treatments, a one‐way analysis of variance (ANOVA) was carried out. Using the same statistical program, post‐hoc comparisons of treatment means were carried out using Duncan's Multiple Range Test (DMRT). Every value was shown as Mean ± Standard Error of the Mean (SEM), and *p* < 0.05 and *p* < 0.01 were regarded as statistically significant. With Graph Pad Prism, graphical representations of the data were created.

## 3 Results

### 3.1 Body Weight

**FIGURE 1:**
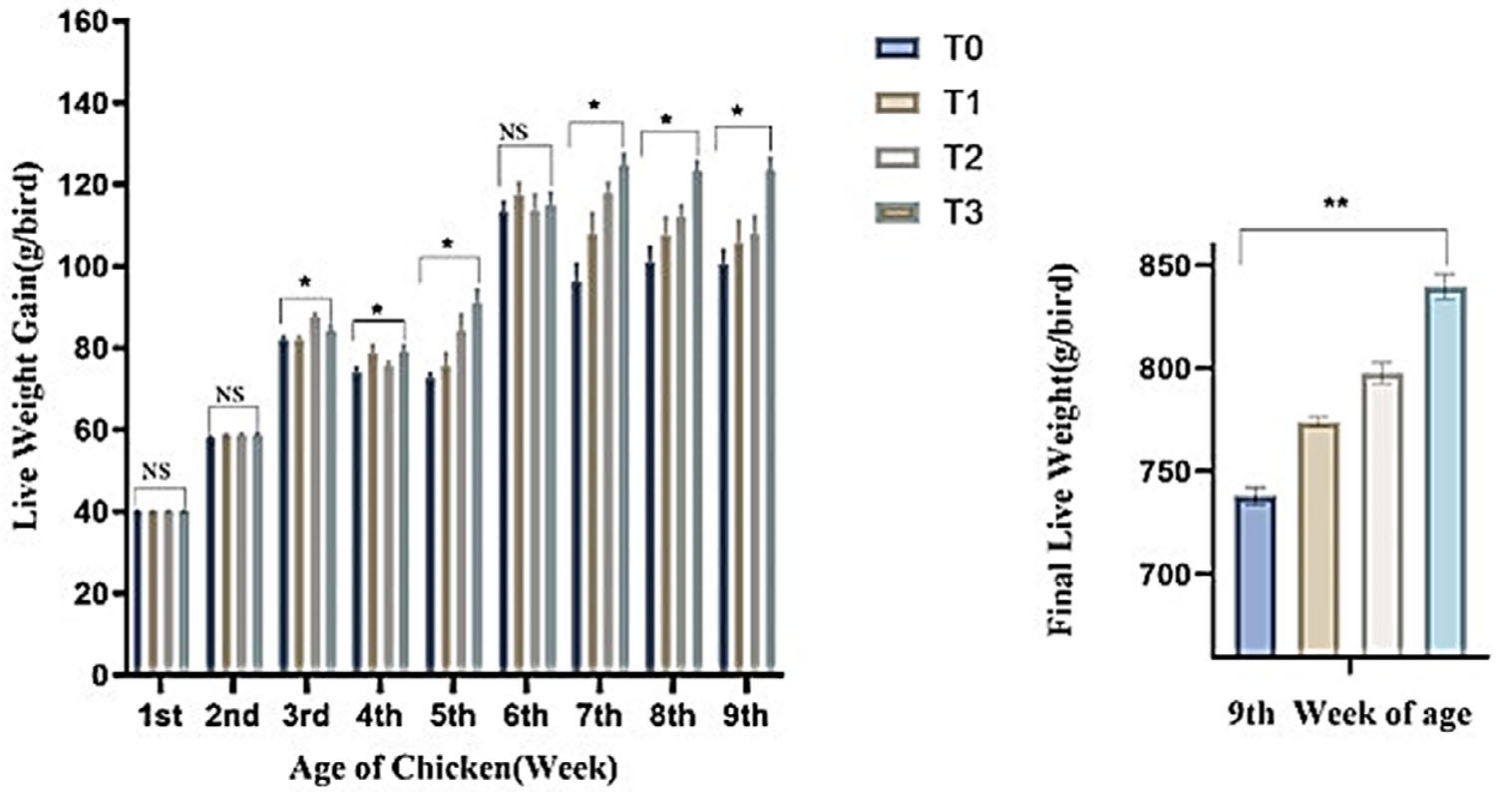
Effect of Shatamuli and Ashwagandha root powder on live weight gain (g/bird) in different treatment groups of Sonali chicken. Values are expressed as mean ± standard error of means (SEM). NS: Statistically not significant (*p* > 0.05). *Indicates 5% level of significance. **indicates 1% level of significance. Here, T_0_ = Diet containing 0% Shatamuli and Ashwagandha root powder, T_1_ = Diet containing 0.5% Ashwagandha root powder, T_2_ = Diet containing 1% Shatamuli root powder, T_3_ = Diet containing 0.5% Shatamuli and 0.5% Ashwagandha root powder.

Figure 1 illustrates the influence of Shatamuli and Ashwagandha root powder supplementation on body weight gain (g/bird) across various treatment groups of Sonali chickens. This figure showed no significant differences at the experiment's first, second and sixth weeks. However, from the third to the fifth and the seventh to the ninth weeks of the trial, the body weight gain varied significantly within the experimental groups. In the Shatamuli and Ashwagandha‐treated group, T_3_ displayed the highest live weight gain (839.64 ± 6.15 g/bird), and the control group, T_0_ (738.10 ± 4.22 g/bird), showed the lowest weight gain. Finally, group T_3_ showed the highest body weight (872.17 ± 6.18 g/bird) compared to the other groups, T_2_ (830.21 ± 5.26 g/bird), T_1_ (806.65 ± 2.17 g/bird) and T_0_ (770.64 ± 4.19 g/bird) respectively.

### 3.2 Feed Intake

**FIGURE 2:**
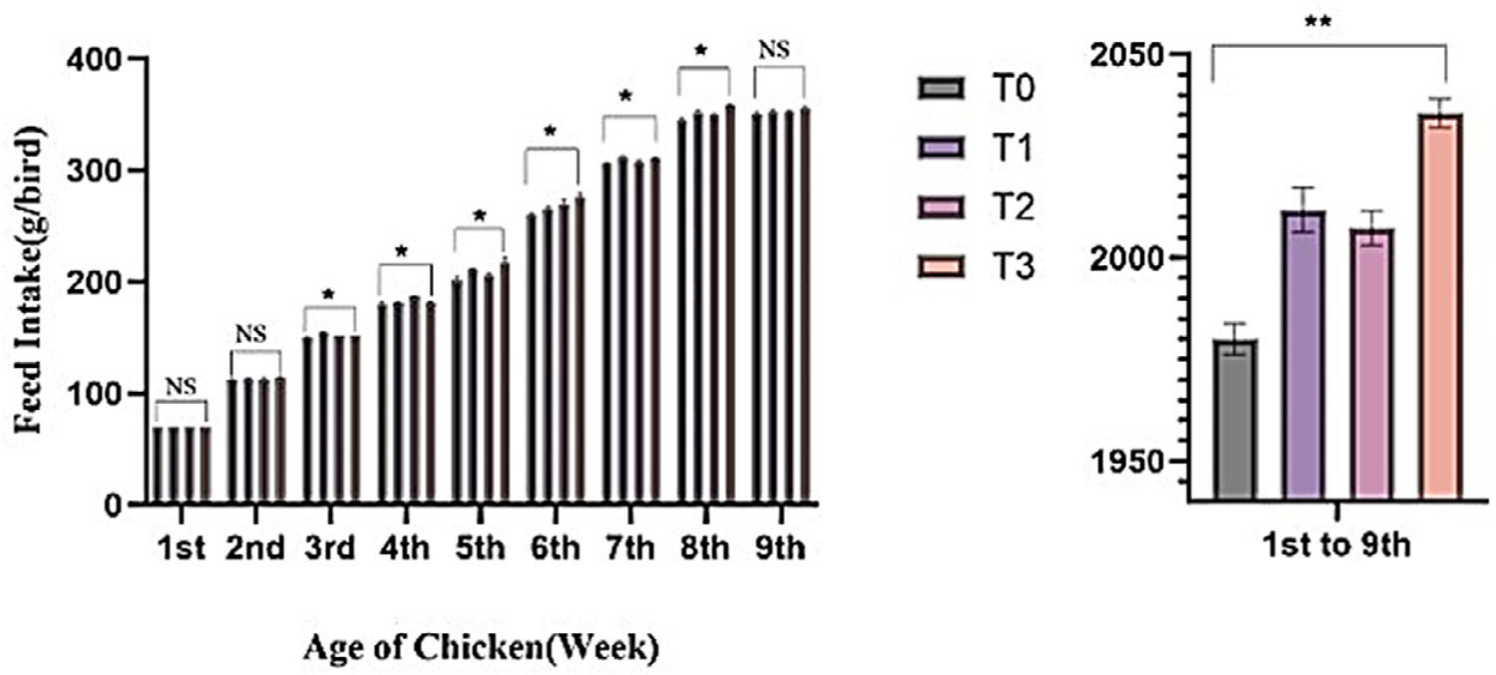
Effect of Shatamuli and Ashwagandha root powder on feed intake (g/bird) in different treatment groups of Sonali chicken. Values are expressed as mean ± standard error of means (SEM). NS: Statistically not significant (*p *> 0.05). *Indicates 5% level of significance. **indicates 1% level of significance. Here, T_0_ = Diet containing 0% Shatamuli and Ashwagandha root powder, T_1_ = Diet containing 0.5% Ashwagandha root powder, T_2_ = Diet containing 1% Shatamuli root powder, T_3_ = Diet containing 0.5% Shatamuli and 0.5% Ashwagandha root powder.

Figure 2 depicts the mean weekly FI of the experimental birds. During the first, second and ninth weeks, the experimental groups showed no significant (*p* > 0.05) changes in feed consumption. In contrast, from the third to eighth weeks, feed consumption varied significantly (*p* < 0.05) across the treatments. Birds receiving Shatamuli and Ashwagandha root powder showed greater FI than the control group. For the entire trial period, the T_3_ group had considerably higher feed consumption (*p* < 0.01) reaching 2035.62 ± 3.55 g/bird. This was followed by T_1_ (2011.83 ± 5.48 g/bird) and T_2_ (2007.31 ± 4.26 g/bird), while the control group (T_0_) recorded the lowest intake (1980.32 ± 3.81 g/bird).

### 3.3 Feed Conversion Ratio (FCR)

**FIGURE 3:**
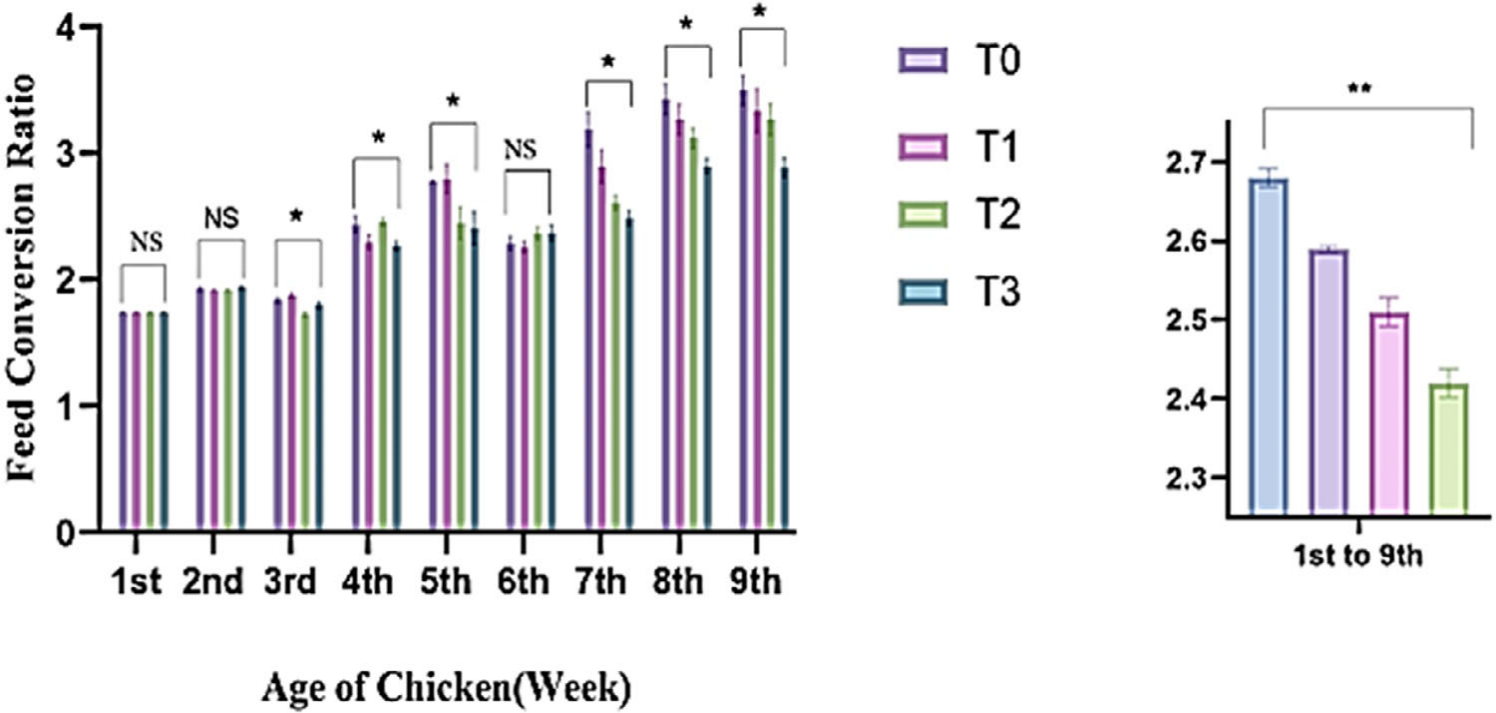
Effect of Shatamuli and Ashwagandha root powder on Feed Conversion Ratio (FCR) in different treatment groups of Sonali chicken. Values are expressed as mean ± standard error of means (SEM). NS: Statistically not significant (*p* > 0.05). *Indicates 5% level of significance. **indicates 1% level of significance. Here, T_0_ = Diet containing 0% Shatamuli and Ashwagandha root powder, T_1_ = Diet containing 0.5% Ashwagandha root powder, T_2_ = Diet containing 1% Shatamuli root powder, T_3_ = Diet containing 0.5% Shatamuli and 0.5% Ashwagandha root powder.

Figure 3 represents the feed efficiency of birds across different treatment groups. During the first, second, and sixth weeks of the experiment, feed efficiency was found to be inconsequential (*p* > 0.05) across all treated groups. However, notable variations (*p* < 0.05) in feed efficiency were calculated during the third to fifth weeks and again from the seventh to ninth weeks across the dietary treatments. Throughout the first to ninth weeks, birds treated with Shatamuli and Ashwagandha root powder demonstrated enhanced feed efficiency relative to the control group. The T_3_ group demonstrated the highest feed‐to‐meat conversion efficiency (2.42 ± 0.018), followed by the T_2_ (2.51 ± 0.018), T_1_ (2.59 ± 0.004) and T_0_ (2.68 ± 0.012) groups. The T_3_ group resulted in the most favourable feed efficiency in comparison to the control and other treated groups.

### 3.4 Carcass Characteristics

Table 3 shows that the combined Shatamuli and Ashwagandha root powder supplied group (T_3_) had the highest live weight (899.56 ± 7.56) compared to the others, T_2_ (843.88 ± 8.78), T_1_ (808.27 ± 9.57) and T_0_ (780.20 ± 15.5), respectively. The T_3_ group showed significantly (*p* < 0.01) the highest carcass weight (559.30 ± 5.99), whereas the lowest was in the control group, T_0_ (475.23 ± 10.01). Dressing percentage was varied significantly (*p* < 0.05), with the highest being in T_3_ (62.17 ± 0.14) and the lowest in the T_0_ group (60.90 ± 0.06). Breast weight, thigh muscle weight and drumstick weight significantly differed among the experimental groups. Abdominal fat, liver, heart, wings and gizzard weights did not vary substantially (*p* > 0.05) across the experimental groups. The pH of meat varied non‐significantly within the experimental groups, and the highest drip loss percentage was found in T_0_ (2.37 ± 0.115) and the lowest in T_3_ (2.24 ± 0.008) group after 24 h of refrigeration.

**TABLE 3:** Impact of Shatamuli and Ashwagandha root powder inclusion on carcass yield parameters in Sonali chickens.

Carcass yield (g)	Treatment groups				*p*‐Value and level of significance
	T_0_	T_1_	T_2_	T_3_	
Live weight (g)	780.20 ± 15.57^a^	808.27 ± 9.57^a^	843.88 ± 8.78^b^	899.56 ± 7.56^c^	<0.001 (**)
Carcass weight (g)	475.23 ± 10.01^a^	495.40 ± 8.14^a^	521.05 ± 6.02^b^	559.30 ± 5.996^c^	<0.001 (**)
Dressing (%)	60.90 ± 0.06^a^	61.28 ± 0.36^ab^	61.74 ± 0.07^bc^	62.17 ± 0.14^c^	0.011 (*)
Breast weight (g)	104.33 ± 2.33^a^	108.26 ± 2.64^ab^	116.67 ± 2.72^bc^	123.67 ± 5.23^c^	0.016 (*)
Thigh muscle weight (g)	70.33 ± 1.45^a^	72.67 ± 1.52^a^	81.33 ± 2.02^b^	86.45 ± 4.72^b^	0.011 (*)
Drumstick weight (g)	62.67 ± 1.73^a^	64.33 ± 2.18^a^	67.33 ± 0.88^ab^	72.67 ± 1.73^b^	0.016 (*)
Abdominal fat (g)	14.67 ± 1.76	13.33 ± 1.45	13.00 ± 1.15	11.33 ± 0.66	0.331 (NS)
Liver weight (g)	24.00 ± 1.15	25.33 ± 0.88	25.67 ± 1.15	26.67 ± 1.45	0.226 (NS)
Heart weight (g)	5.67 ± 0.66	6.33 ± 0.57	7.00 ± 0.57	7.67 ± 0.88	0.421 (NS)
Wings (g)	24.67 ± 1.00	25.33 ± 0.57	25.67 ± 0.66	27.33 ± 0.88	0.223 (NS)
Gizzard (g)	19.67 ± 0.88	22.67 ± 1.00	24.33 ± 2.30	26.33 ± 2.33	0.941(NS)
pH	6.15 ± 0.066	5.95 ± 0.014	5.91 ± 0.088	5.87 ± 0.029	0.223 (NS)
Drip loss% (after 24 h)	2.37 ± 0.115	2.30 ± 0.011	2.29 ± 0.029	2.24 ± 0.008	0.548 (NS)

*Note*: All values are expressed as the mean along with their standard error (SEM). The term NS indicates that differences were not statistically significant (*p* > 0.05). Means in the same row that carry different superscript letters (a, b, c) signify significant variation among treatments at *p* < 0.05. A single asterisk (*) denotes significance at the 5% level, whereas a double asterisk (**) denotes significance at the 1% level.

The treatment groups were formulated as follows,

T_0_—Control diet without the inclusion of Shatamuli or Ashwagandha root powder.

T_1_—Diet supplemented with 0.5% Ashwagandha root powder.

T_2_—Diet supplemented with 1% Shatamuli root powder.

T_3_—Diet supplemented with 0.5% Shatamuli and 0.5% Ashwagandha root powder.

### 3.5 Haematological and Lipid Profile Parameters

Table 4 displays how the powder form of Ashwagandha and Shatamuli roots affected the experimental birds' blood profiles. It showed that the total cholesterol (mg/dL) level was varied notably (*p* < 0.05) among the experimental groups, and the lowest was observed in group T_3_ (114.08 ± 9.56), and the highest was in the control group, T_0_ (162.06 ± 3.11), whereas T_2_ (135.27 ± 6.84) and T_1_ (150.54 ± 7.78), respectively. The lowest LDL (mg/dL) level was found in group T_3_ (66.37 ± 3.35) compared to the other groups, T_0_ (81.05 ± 14.33), T_1_ (73.26 ± 3.05), and T_2_ (71.83 ± 9.27), respectively. The highest HDL level (mg/dL) was found in the combined treated group T_3_ (54.57 ± 2.86), and the lowest was in the T_0_ group (37.03 ± 2.23). Table 4 demonstrates that Triglyceride (mg/dL), haemoglobin (g/dL), RBC (cells 10^6^/µL), packed cell volume (PCV%), erythrocyte sedimentation rate (ESR) and mean corpuscular volume, MCV (fL), WBC (cells 10^3^/µL) did not differ substantially (*p* > 0.05) within the groups. Haemoglobin (Hb) was highest in the T_3_ (11.96 ± 0.23) group and lowest in the T_0_ (10.73 ± 1.53) group. The T_3_ group also demonstrated significantly (*p* < 0.05) reduced glucose level (189.87 ± 7.43) compared to control, T_0_ (233.67 ± 3.99), and other groups, T_1_ (228.02 ± 3.58) and T_2_ (214.03 ± 9.32), respectively.

**TABLE 4:** Effect of Shatamuli and Ashwagandha root powder on lipid profile and haematological parameters of Sonali chickens.

Parameters	Treatment groups				*p*‐Value and level of significance
	T_0_	T_1_	T_2_	T_3_	
Total cholesterol (mg/dL)	162.06 ± 3.11^c^	150.54 ± 7.78^bc^	135.27 ± 6.84^ab^	114.08 ± 9.65^a^	0.008 (*)
Triglyceride (mg/dL)	75.52 ± 3.71	64.83 ± 3.44	58.59 ± 10.28	62.81 ± 6.92	0.383 (NS)
HDL (mg/dL)	37.03 ± 2.23	41.08 ± 0.63	43.77 ± 6.51	54.57 ± 2.86	0.052 (NS)
LDL (mg/dL)	81.05 ± 14.33	73.26 ± 3.05	71.83 ± 9.27	66.37 ± 3.35	0.711 (NS)
Hb (g/dL)	10.73 ± 1.53	11.43 ± 0.53	11.53 ± 0.61	11.96 ± 0.23	0.797 (NS)
RBC (cells 10^6^/µL)	2.49 ± 0.09	2.51 ± 0.10	2.55 ± 0.13	2.64 ± 0.07	0.737 (NS)
PCV (%)	28.80 ± 1.57	28.60 ± 1.64	29.98 ± 1.33	31.64 ± 1.62	0.521 (NS)
ESR (mm/1st hour)	1.66 ± 0.18	1.29 ± 0.16	1.49 ± 0.11	1.30 ± 0.17	0.371 (NS)
MCV (fL)	134.24 ± 2.89	136.83 ± 1.16	137.61 ± 0.53	136.70 ± 1.01	0.147 (NS)
WBC (cells 10^3^/µL)	2.74 ± 0.08	2.83 ± 0.43	2.63 ± 0.03	2.57 ± 0.04	0.842 (NS)
Glucose (mg/dL)	233.67 ± 3.99^b^	228.02 ± 3.58^b^	214.03 ± 9.3^b^	189.87 ± 7.43^a^	0.006 (*)

*Note*: All values are expressed as the mean along with their standard error (SEM). The term NS indicates that differences were not statistically significant (*p* > 0.05). Means in the same row that carry different superscript letters (a, b, c) signify significant variation among treatments at *p* < 0.05. A single asterisk (*) denotes significance at the 5% level, whereas a double asterisk (**) denotes significance at the 1% level.

The treatment groups were formulated as follows,

T_0_—Control diet without the inclusion of Shatamuli or Ashwagandha root powder.

T_1_—Diet supplemented with 0.5% Ashwagandha root powder.

T_2_—Diet supplemented with 1% Shatamuli root powder.

T_3_—Diet supplemented with 0.5% Shatamuli and 0.5% Ashwagandha root powder.

### 3.6 Faecal Microbial Loads

**FIGURE 4:**
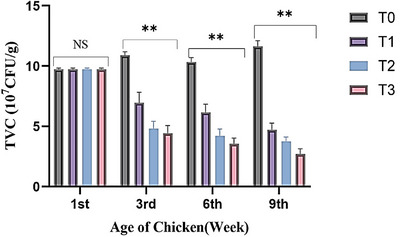
Effect of Shatamuli and Ashwagandha root powder on faecal total viable counts (10^7^CFU/g) of Sonali chicken. Values are expressed as mean ± standard error of means (SEM). NS: Statistically not significant (*p* > 0.05). **indicates 1% level of significance. Here, T_0_ = Diet containing 0% Shatamuli and Ashwagandha root powder, T_1_ = Diet containing 0.5% Ashwagandha root powder, T_2_ = Diet containing 1% Shatamuli root powder, T_3_ = Diet containing 0.5% Shatamuli and 0.5% Ashwagandha root powder.

Figure 4 demonstrates the consequence of Ashwagandha and Shatamuli root powder on the faecal total viable count (10^7^CFU/g). The total faecal viable count at the experiment's third, sixth and ninth weeks was notably lower (*p* < 0.01) in the treatment groups than in the control group. In the first week, before initiation of the treatment, the TVC (9.73 ± 0.12) was not varied significantly (*p* > 0.05) in all experimental birds. The TVC in the control group increases with the advancement of age; on the contrary, there was a marked (*p* < 0.01) reduction of faecal microbial loads in the treated groups T_1_ (4.71 ± 0.56), T_2_ (3.76 ± 0.36) and T_3_ (2.70 ± 0.44), respectively, after the ninth week of treatment.

### 3.7 Cost Effectiveness of Production

Table 5 outlines the production costs for Sonali chicks supplemented with Shatamuli and Ashwagandha root powder. Expenses including feed, chicks, vaccines, medications, litter, rena C, Shatamuli and Ashwagandha root powder, PPM solution and miscellaneous costs (labour, electricity and transportation), which were calculated as cost per chick and cost per kg of live weight. At the conclusion of the study, total production costs per bird showed significant variation (*p* < 0.01) across the treatment groups. The total production costs ($/bird) were recorded as follows: T_0_ (1.76 ± 0.002), T_1_ (1.84 ± 0.003), T_2_ (1.86 ±  0.002) and T_3_ (1.89 ± 0.002). Net profit per bird ($) also varied markedly (*p* < 0.01) among the groups, with the T_3_ group yielding the highest net profit (0.29 ± 0.014), followed by T_2_ (0.21 ± 0.014), T_1_ (0.18 ± 0.003), and the lowest in T_0_ (0.16 ± 0.009).

**TABLE 5:** Cost effectiveness of Sonali chicken production using Shatamuli and Ashwagandha as a feed additive.

Parameters	Treatment groups				*p*‐Value and level of significance
	T_0_	T_1_	T_2_	T_3_	
Cost/Chick ($)	0.32 ± 0.00	0.32 ± 0.00	0.32 ± 0.00	0.32 ± 0.00	NS
Average feed consumption (kg/Bird)	1.98 ± 0.003^a^	2.01 ± 0.005^b^	2.01 ± 0.004^b^	2.03 ± 0.003^c^	<0.001 (**)
Feed price/kg ($)	0.55 ± 0.00	0.55 ± 0.00	0.55 ± 0.00	0.55 ± 0.00	NS
Cost of Shatamuli and Ashwagandha ($/Sonali)	0.00 ± 0.00	0.06 ± 0.00	0.08 ± 0.00	0.10 ± 0.00	NS
Total Feed Cost/Sonali ($)	1.08 ± 0.002^a^	1.11 ± 0.003^b^	1.10 ± 0.002^b^	1.12 ± 0.002^c^	<0.001 (**)
Miscellaneous ($)	0.36 ± 0.00	0.36 ± 0.00	0.36 ± 0.00	0.36 ± 0.00	NS
Total cost/Sonali ($)	1.76 ± 0.002^a^	1.84 ± 0.003^b^	1.86 ± 0.002^c^	1.89 ± 0.002^d^	<0.001 (**)
Avg. Live weight (kg)	0.770 ± 0.004^a^	0.806 ± 0.002^b^	0.830 ± 0.005^c^	0.872 ± 0.006^d^	<0.001 (**)
Sales price/kg ($)	2.50 ± 0.00	2.50 ± 0.00	2.50 ± 0.00	2.50 ± 0.00	NS
Sales price/Sonali ($)	1.93 ± 0.01^a^	2.02 ± 0.005^b^	2.07 ± 0.013^c^	2.18 ± 0.015^d^	<0.001 (**)
Net profit/Sonali ($)	0.16 ± 0.009^a^	0.18 ± 0.003^a^	0.21 ± 0.014^b^	0.29 ± 0.014^c^	<0.001 (**)

*Note*: All values are expressed as the mean along with their standard error (SEM). The term NS indicates that differences were not statistically significant (*p* > 0.05). Means in the same row that carry different superscript letters (a, b, c) signify significant variation among treatments at *p* < 0.05. A single asterisk (*) denotes significance at the 5% level, whereas a double asterisk (**) denotes significance at the 1% level.

The treatment groups were formulated as follows,

T_0_—Control diet without the inclusion of Shatamuli or Ashwagandha root powder.

T_1_—Diet supplemented with 0.5% Ashwagandha root powder.

T_2_—Diet supplemented with 1% Shatamuli root powder.

T_3_—Diet supplemented with 0.5% Shatamuli and 0.5% Ashwagandha root powder.

## 4 Discussion

The study results indicated that groups supplemented with Shatamuli and Ashwagandha root powder exhibited significantly higher (*p* < 0.01) live weight and live weight gain relative to the control group. The T_3_ group, receiving 0.5% Shatamuli and Ashwagandha powder, recorded the highest body weight and weight gain, while the control group showed the lowest. These results align with Nagar et al. (2021), who observed that a diet supplemented with 2.5 g Shatamuli and 2.5 g Ashwagandha per kg of feed enhanced body weight and weight gain in caged broilers compared to their individual use. Similarly, Adangle et al. (2017) reported improved growth performance in Giriraja poultry supplemented with Shatamuli and Ashwagandha compared to the control group. Enhanced growth rates in groups supplemented with *W. somnifera* (Ashwagandha) root powder were also noted by Jyotsana et al. (2018) and Yadav et al. (2018). Dhenge et al. (2018) found that 0.5% Ashwagandha supplementation was optimal for weekly body weight gain in broilers. Mirjalili et al. (2009) suggested that Ashwagandha's provision of vitamins A, D, E and thiamine enhances bird performance under high temperatures. These results are consistent with Kumar et al. (2019), Patil et al. (2024), Verma et al. (2024) and Yadav et al. (2018), who reported increased body weight with *A. racemosus* (Shatamuli) root powder supplementation at 1% and 1.5% as an herbal growth promoter. Amanullah et al. (2009) attributed significant weight gain differences to higher digestible crude protein (DCP), total digestible nutrients (TDN) and nutrient digestibility (e.g., dry matter and crude protein), along with elevated calcium, zinc and vitamin B content in Shatamuli‐supplemented diets.

Feed consumption varied markedly (*p* < 0.01) in groups provided with Shatamuli and Ashwagandha root powder compared to the control, with the greatest intake noted in the combined supplementation group and the smallest in the control group. These findings are supported by Jediya et al. (2025), Jyotsana et al. (2018) and Nagar et al. (2021), who reported that 2.5 g each of Shatamuli and Ashwagandha per kg of feed improved FI in broilers compared to individual supplementation. Kumar et al. (2019) linked dietary Ashwagandha to increased FI, while Lee et al. (2020) associated it with thyroid stimulation, enhanced metabolism and increased muscle mass in poultry. Jyotsana et al. (2018) noted that 1% Ashwagandha supplementation significantly (*p* < 0.05) enhanced feed consumption, and Jediya et al. (2025) confirmed significant (*p* < 0.01) differences in FI across treatment groups.

In this study, the combined supplementation of Shatamuli and Ashwagandha in Sonali chickens resulted in the minimum FCR and the best feed efficiency compared to other groups. These findings corroborate Nagar et al. (2021), who narrated improved FCR in broilers fed a combination of Shatamuli and Ashwagandha compared to other treatments. Enhanced feed utilization due to Shatamuli supplementation was also reported by Kumar et al. (2019), Patil et al. (2024), Verma et al. (2024) and Yadav et al. (2018). Ashwagandha supplementation promotes metabolism and digestion (Wal et al. 2019) and improves FCR, as noted by Saini et al. (2017), who observed enhanced nutrient digestibility in broilers fed 0.5%–1.5% Ashwagandha. In addition, Jyotsana et al. (2018) found that diets containing 0.5%, 0.75% and 1% Ashwagandha root powder increased intestinal villus formation in broilers.

The overall MR in the study was 3.12%, with the combined Shatamuli and Ashwagandha group recording zero mortality, while the control group had the highest MR. Ahmad (2008) similarly reported reduced mortality in Shatamuli‐supplemented groups. Salem et al. (2022) attributed lower mortality to improved humoral and cell‐mediated immune responses in Ashwagandha‐supplemented broilers.

At the experiment's conclusion, the combined supplementation group exhibited superior carcass characteristics, including higher carcass weight, dressing percentage, breast muscle, thigh, drumstick and reduced abdominal fat, compared to the control and other groups. Bhardwaj et al. (2008) reported improved conformation in quail chicks supplemented with 1% Shatamuli and 1% Ashwagandha root powder compared to controls. Pandey et al. (2013) observed increased dressing and eviscerated weight percentages with up to 1% Shatamuli supplementation, while Dahale et al. (2014) noted enhanced growth performance and carcass quality. According to Gaikwad et al. (2015), incorporating 0.5%–1.5% *W. somnifera* (WS) powder into broiler diets enhances carcass yield, eviscerated carcass weight, thigh and breast percentages and gizzard weight. Srivastava et al. (2013) reported that supplementation with indigenous herbal drugs, including *W. somnifera*, *A. racemosus* and *Mucuna pruriens*, at varying levels increased the weights of the gizzard, liver and heart compared to the control group. Chikwa et al. (2018) noted significantly higher meat yield with diets containing 1% Shatamuli root powder, 1% Ashwagandha root powder and 200 mg/kg of vitamin E.

Supplementation with Shatamuli (*A. racemosus*) and Ashwagandha (*W. somnifera*) root powder significantly affected the haematological and lipid profiles of Sonali chickens. Verma et al. (2024) observed increased Hb and PCV values, with reduced total leukocyte count (TLC) in birds receiving SRP, particularly at 1% inclusion level. Similarly, Biswas et al. (2020) and De Oliveira et al. (2024) reported that Ashwagandha supplementation improved Hb, RBC and WBC counts in broilers. Alok et al. (2013) studied that steroidal saponins and withanolides are responsible for these improvements, which exert antioxidant and haematopoietic effects by protecting erythrocytes from oxidative stress and promoting erythropoiesis. The reduction in serum total cholesterol, triglycerides and glucose levels observed with Shatamuli and Ashwagandha supplementation aligns with their reported hypolipidemic and hypoglycemic properties. Salem et al. (2022) found that these effects are attributed to the modulation of lipid metabolism and antioxidant activity. Visavadiya et al. (2009) reported that Shatamuli root contains phytosterols, saponins, polyphenols, flavonoids and ascorbic acid, which promote bile acid synthesis and cholesterol excretion. Similarly, according to Uthirapathy and Tahir (2021), supplementation with Ashwagandha reduces total cholesterol, triglycerides and LDL levels. In addition, Sarangi et al. (2013) found that Ashwagandha increases the amount of insulin secreted by pancreatic β‐cells, thereby contributing to lower blood glucose concentrations.

The study revealed that birds receiving a combined Shatamuli and Ashwagandha powder diet exhibited significantly (*p* < 0.01) reduced faecal microbial loads with advancing age compared to other groups. These results align with Bokaeian and Saeidi (2015) and Jyotsana et al. (2018), who reported significant reductions in bacterial loads in birds fed diets supplemented with Shatamuli and Ashwagandha. Mandal et al. (2007) investigated Shatamuli's effects on bacteria such as *Escherichia coli*, *Shigella dysenteriae*, *Salmonella typhi* and *Pseudomonas*. Similarly, Kumari and Gupta (2015) highlighted Ashwagandha's antimicrobial properties against bacteria, including *Bacillus subtilis*, *Enterobacter aerogenes*, *Klebsiella pneumoniae*, *Proteus mirabilis*, and *E. coli*.

The study found significant differences (*p* < 0.01) in total production costs per bird among the experimental groups, with the T_3_ group incurring the highest cost but also yielding the highest net profit, while the control group recorded the lowest profit. These findings are consistent with Adangle et al. (2017) and Mane et al. (2012). Nagar et al. (2021) assessed the combined effect of Shatamuli and Ashwagandha root powder in broiler diets, reporting that birds supplemented with both achieved the highest body weight and net profit compared to single supplementation. Pedhavi et al. (2017) noted higher net returns in broilers treated with 20% WS root extract. Gaikwad et al. (2015) undertook an analysis with broiler chicks given Shatamuli root powder at 0%, 0.5% and 1% levels, finding that the 1% level resulted in the highest net profit.

## 5 Conclusions

The experimental results showed that a combination of Shatamuli and Ashwagandha root powder improved carcass characteristics, growth performance and reduced microbial loads compared to groups receiving either herb alone. Blood parameters were also improved more in the combination group. These herbs have synergistic effects as Ashwagandha enhances metabolism and reduces oxidative stress, and Shatamuli supports digestion, immunity and nutrient absorption. Together, they complement each other's mechanisms, amplifying the overall physiological benefits in chickens. Therefore, a combined supplementation of Shatamuli and Ashwagandha root powder may be used to enhance performance at Sonali chicken farm. However, further study is necessary to determine the combined dose for optimal output.

## Author Contributions

**Ayesha Siddika**: data collection, data analysis, laboratory test, writing – original draft. **Md. Ahsan Habib**: study's conception, methodology, layout design, laboratory test, supervision, writing – original draft. **Sabbir Hossen Sabuz**: supervision, investigation, data curation. **Ummay Salma**: supervision, validation, funding acquisition. **Md. Nurul Amin**: investigation, ethical approval, data analysis**. Md Abdur Rahman Toha**: editing original draft, data analysis.

## Funding

This study was supported by the Ministry of Science and Technology (MoST), Bangladesh (NST Fellowship MS/MSc 2023/2024, Merit No. 461; Registration No.917).

## Ethics Statement

All procedures involving birds were approved by the Department of Animal Science and Nutrition, Faculty of Veterinary and Animal Science, Hajee Mohammad Danesh Science and Technology University, Dinajpur‐5200, Bangladesh (Approval No. HSTU/VAS/ASN/EA/020, Date: 13/05/2024).

## Conflicts of Interest

The authors declare no conflicts of interest.

## Data Availability

The data supporting the findings of this study are available from the corresponding author upon reasonable request.
